# Polycystic Disease of the Salivary Glands: A Rare Cause of Bilateral Parotid Enlargement

**DOI:** 10.7759/cureus.97591

**Published:** 2025-11-23

**Authors:** Aeimen Khalid, Muhammad Ahmad Mukhtar, Amna Mukhtar, Rubina Mukhtar

**Affiliations:** 1 General Medicine, Peterborough City Hospital, Peterborough, GBR; 2 General Medicine, York District Hospital, York, GBR; 3 Radiology, Nishtar Medical University, Multan, PAK; 4 Internal Medicine, University of Debrecen, Debrecen, HUN; 5 Radiology, Multan Institute of Nuclear Medicine and Radiotherapy (MINAR) Cancer Hospital, Multan, PAK

**Keywords:** cysts, dysgenetic polycystic disease (dpd), histopathology, mri, neoplastic, parotid glands, salivary glands

## Abstract

Non-neoplastic cysts are rare, causing salivary gland enlargement. Among these, one of the rarest entities is dysgenetic polycystic disease (DPD) of the salivary glands. Also referred to simply as polycystic disease, DPD is a rare developmental anomaly of the salivary gland ductal system, characterized by distinctive histopathological features. It is an uncommon cause of parotid gland enlargement, with only 22 cases documented in the literature to date. We present a case of DPD in a female patient who exhibited bilateral enlargement of the parotid glands. The diagnosis was confirmed using magnetic resonance imaging (MRI), and the patient was referred to a surgeon who opted for a total parotidectomy.

## Introduction

Salivary gland cystic lesions encompass a wide range of both neoplastic and non-neoplastic conditions [1]. The considerable overlap in their histopathological characteristics poses a diagnostic challenge for both histopathologists and surgeons, making precise diagnosis and effective treatment planning more difficult.

Dysgenetic polycystic disease (DPD) of the parotid glands is a rare condition first described by Seifert et al. in 1981 [2,3]. However, a thorough review indicates that histological confirmation of the disease has been documented in only 22 cases [4]. In earlier reported cases, the common presentation was either as an incidental discovery or as a bilateral swelling of the parotid glands and, in rare instances, involving both submandibular glands. Parotid gland enlargement is typically painless and can persist for long durations; in many cases, recurrent swelling begins in childhood, though noticeable symptoms may not appear until adulthood [5].

The condition shows a female predominance, with a male-to-female ratio of 6:13 [5]. It primarily affects the parotid glands, accounting for 74% of reported cases, of which nine involved bilateral parotid enlargement and five were confined to the right parotid gland [2].

The condition is recognized as familial, with one documented case involving both a mother and her daughter. Additionally, one of the two cases reported by the Armed Forces Institute of Pathology was observed in a male patient [6]. The disease is thought to be inherited and potentially linked to sex chromosomes. Typically, sex-linked traits are observed primarily in males [7]. No known association exists between this condition and polycystic disease affecting other organ systems, such as the kidneys or pancreas.

The lesion is usually confined to a single lobule of the salivary gland and generally shows no clinical or histological evidence of inflammation. It is believed to result from a developmental anomaly affecting the distal duct system [5].

The range of differential diagnoses for bilateral parotid swelling is relatively narrow. The primary considerations include sialodochitis or chronic sialectasis. Although diffuse, infiltrative malignancies like lymphoma or leukemia could be potential diagnoses, they are uncommon in the absence of additional clinical signs or systemic symptoms. This condition is characterized by a number of unique clinical features [8].

## Case presentation

A 27-year-old woman presented with bilateral, painless parotid gland swelling for a few days. She reported a longstanding history of recurrent "swollen glands" dating back to childhood. In the past, these episodes were milder and resolved spontaneously, unlike the current, more pronounced swelling. On physical examination, there was a diffuse and uniform enlargement of the parotid glands on both sides. The swellings were tender to touch and firm in texture and exhibited fluctuation. The overlying skin appeared healthy and was freely mobile, with no attachment to deeper tissues. No regional lymph nodes were palpable, and facial nerve function was preserved bilaterally.

**Figure 1:**
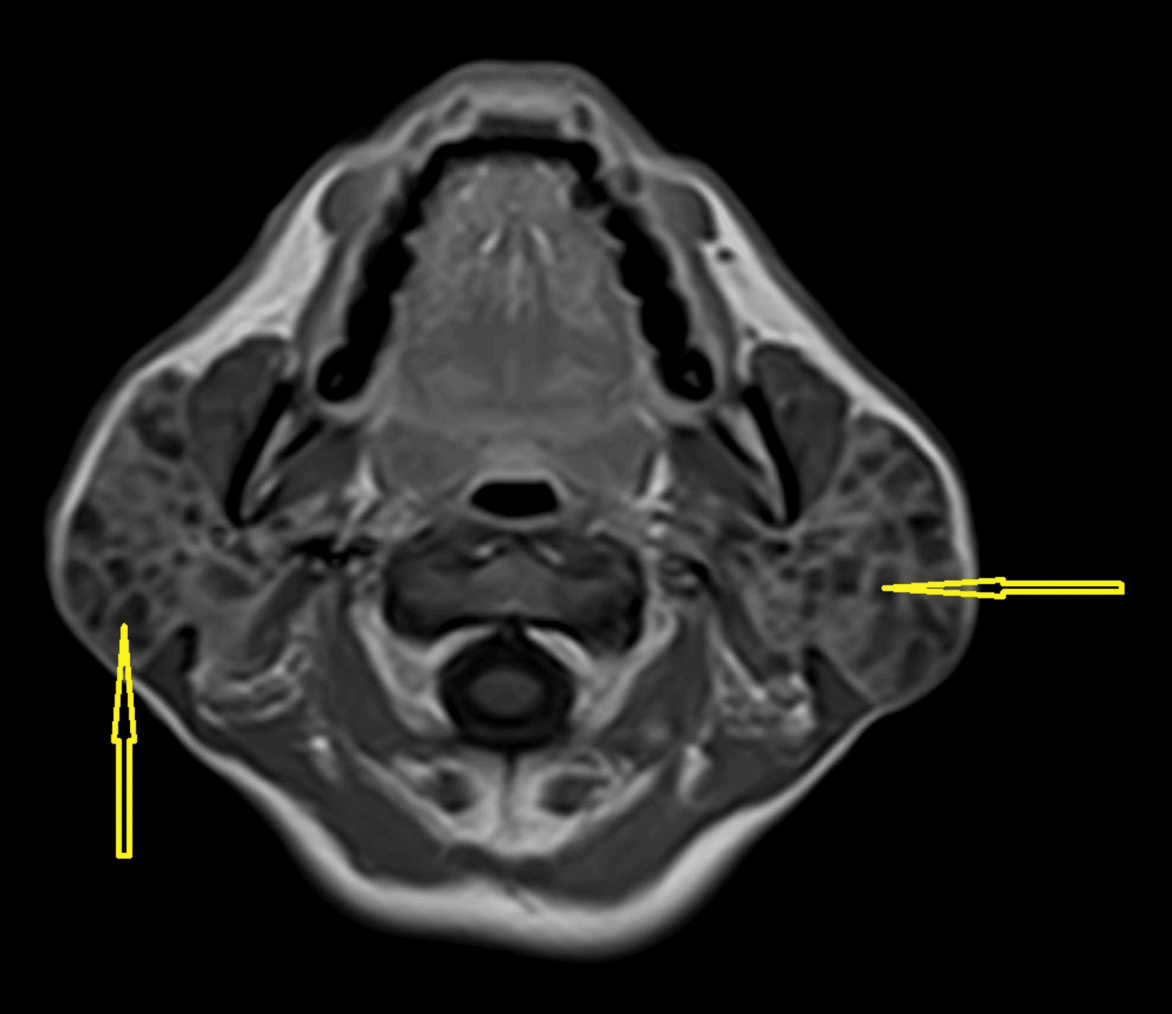
Contrast-enhanced T1-weighted axial image showing the hypointense lesions in the bilateral enlarged parotid glands

**Figure 2:**
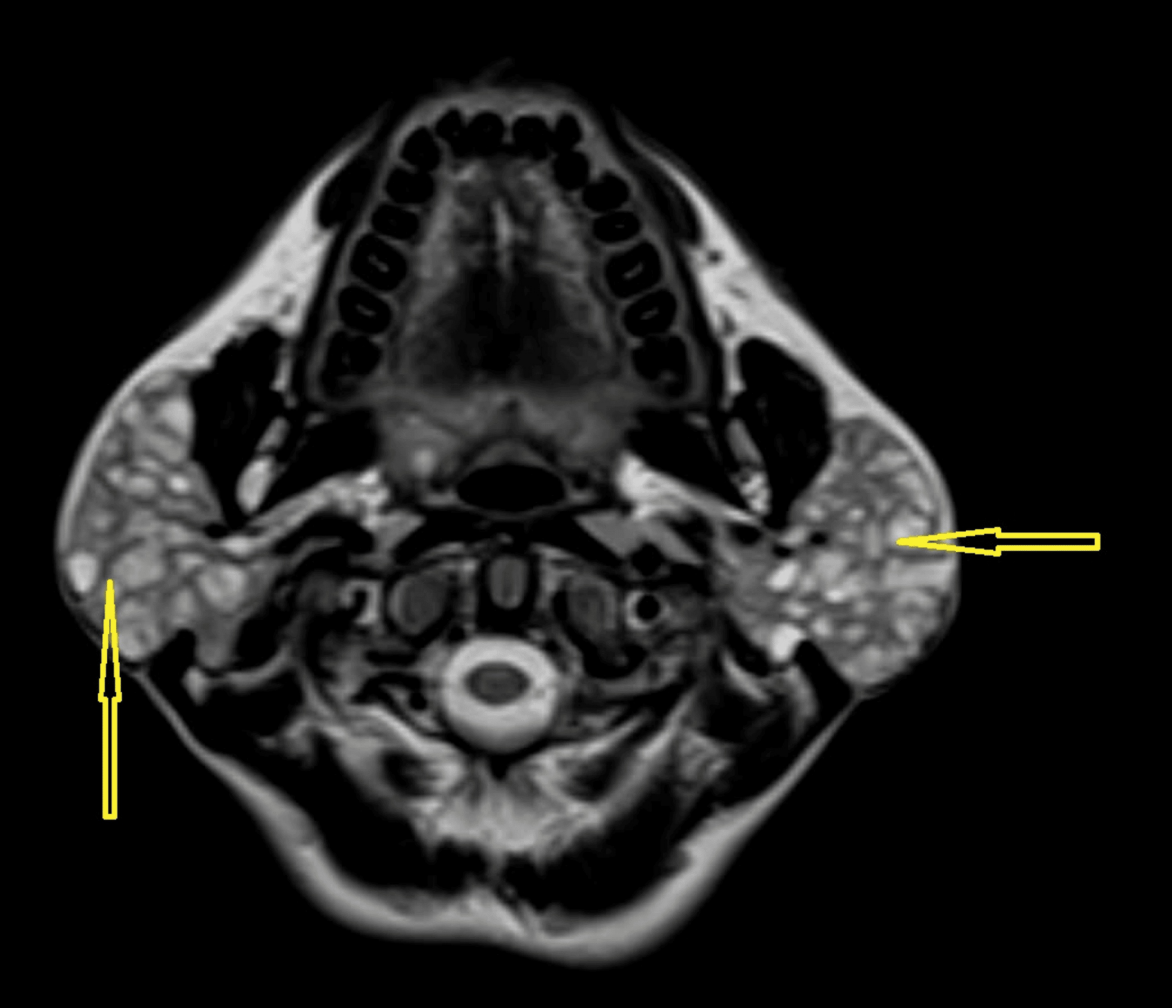
T2-weighted axial image showing the hyperintense lesions in the bilateral enlarged parotid glands

**Figure 3:**
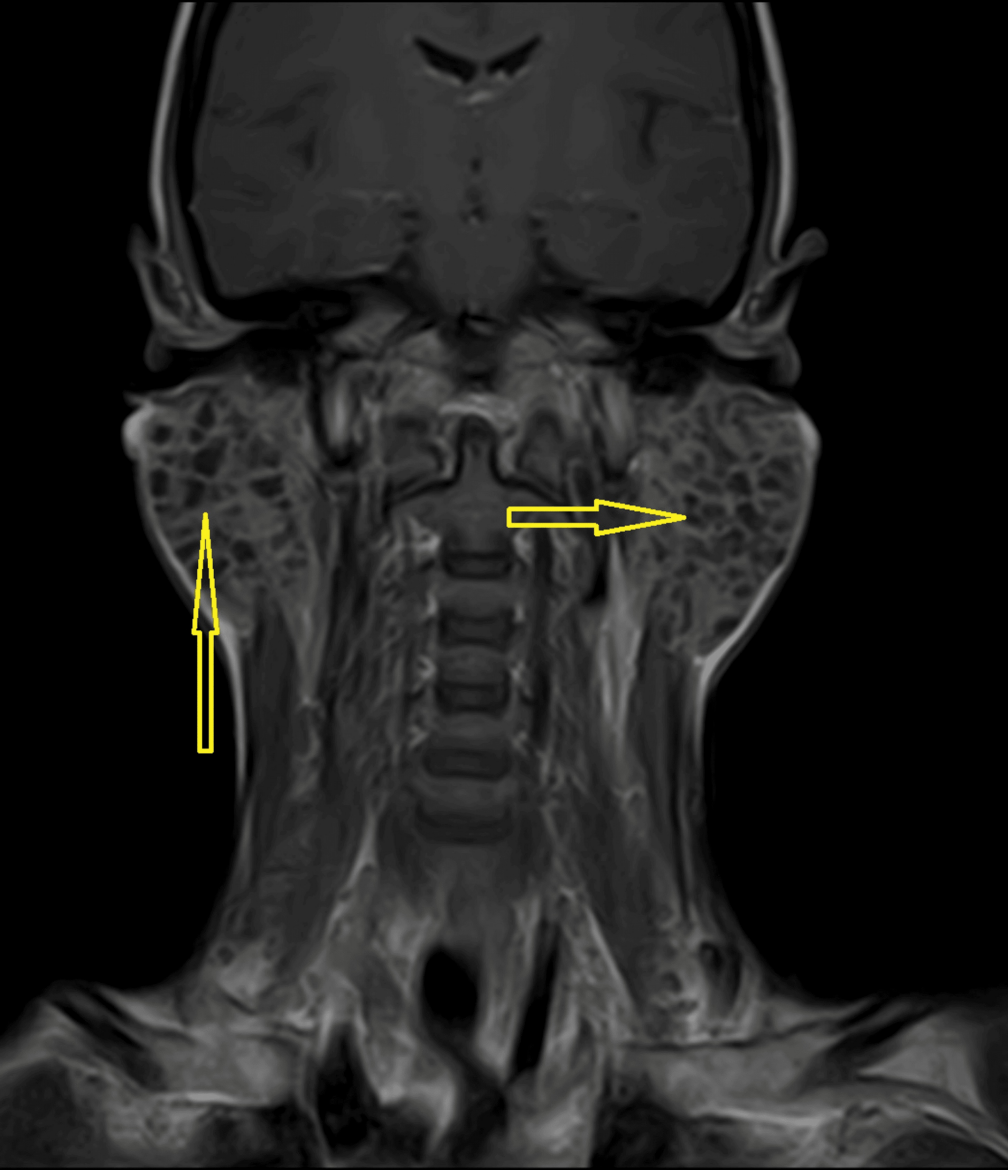
Contrast-enhanced T1-weighted coronal image showing the hypointense lesions in the bilateral enlarged parotid glands

**Figure 4:**
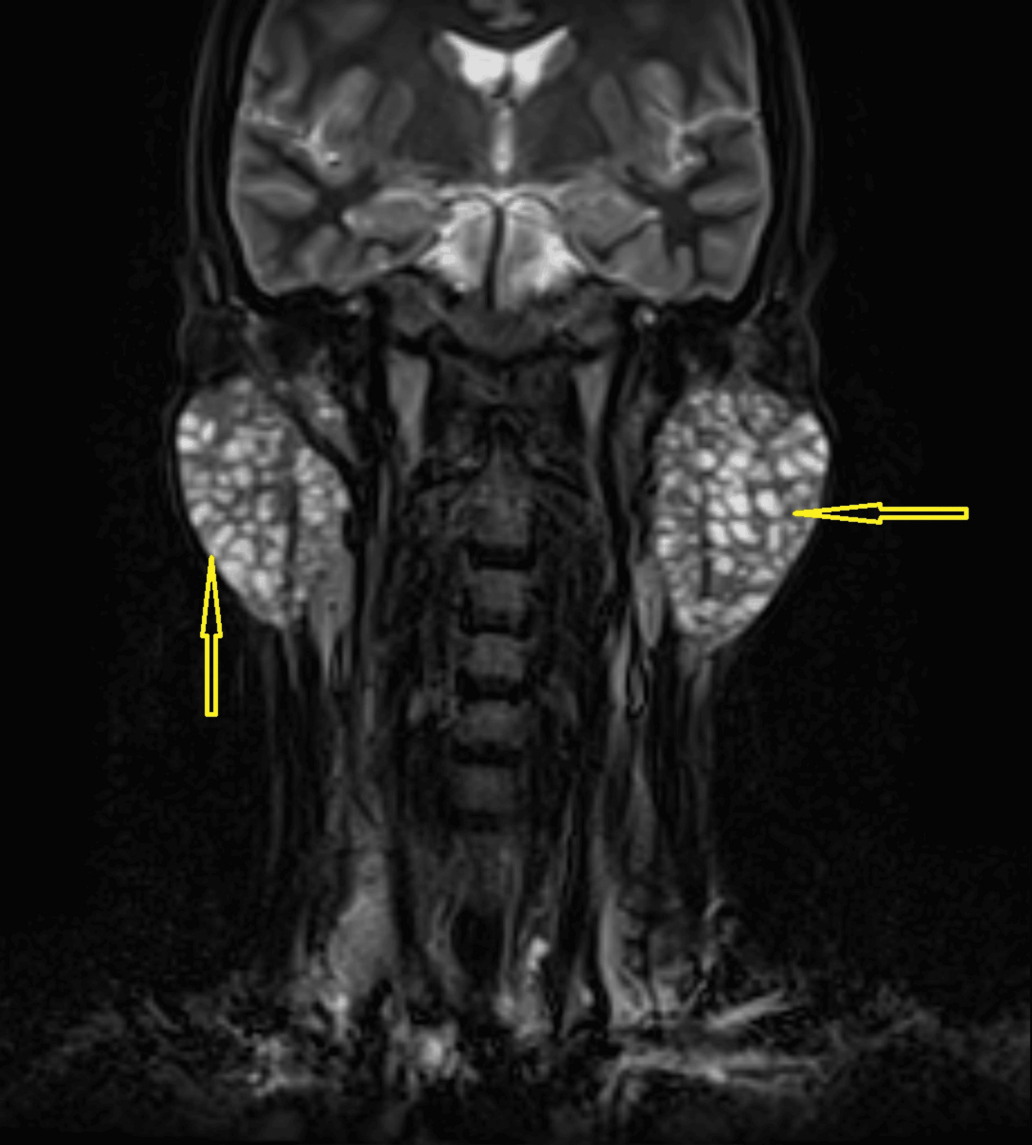
T2-weighted coronal image showing the hyperintense lesions in the bilateral enlarged parotid glands

Salivary flow from Stenson's ducts was clear on both sides. The chest X-ray showed no abnormalities. Routine laboratory tests, including complete blood count (CBC), liver function tests (LFTs), and random blood glucose (RBG), were within normal limits as shown in Table 1. Abdominal ultrasound indicated that all abdominal organs appeared normal. A preliminary clinical diagnosis suggested a benign cystic neoplasm. Magnetic resonance imaging (MRI) demonstrated bilateral enlarged parotid glands. Multiple small cystic areas with a size range of 3 mm to 1 cm were observed, appearing as hypointense lesions on contrast-enhanced T1-weighted images (Figure 1 and Figure 3) and hyperintense on T2-weighted sequences (Figure 2 and Figure 4). The gland appeared well-defined, with no signs of invasion into adjacent tissues, consistent with a benign cystic lesion. Based on the clinical findings, imaging results, and presence of multiple cystic areas within the gland, the possibility of malignancy was ruled out. The patient was subsequently referred to a surgeon, who opted to proceed with a total parotidectomy while carefully preserving the facial nerve. The excised specimen was submitted for histopathological evaluation, which revealed numerous cystic spaces of varying sizes, from a few millimeters to several centimeters in diameter, embedded within a loose, myxoid, and bland connective tissue stroma. The normal glandular parenchyma was entirely replaced by these cystic structures and surrounding connective tissue. The cystic cavities were lined with diverse epithelial cell types, including cuboidal, columnar, and squamous cells, and were filled with watery to pale eosinophilic material.

**Table 1:** Routine laboratory test reports CBC: complete blood count; Hb: hemoglobin; TLC: total leukocyte count; LFTs: liver function tests; SGOT: serum glutamic-oxaloacetic transaminase; SGPT: serum glutamic pyruvic transaminase; RFTs: renal function tests

Sr. no.	Test		Report	Normal values
1	CBC	Hb	12.5 g/dL	11.5-16.5 g/dL
		TLC	6600/ul	4000-11000/ul
		Platelet count	264×10^3^/ul	100-400×10^3^/ul
		Neutrophils	68%	28-78%
		Lymphocytes	24%	17-57%
		Monocytes	7%	<10%
		Basophils	0.9%	<2%
		Eosinophils	1.1%	<10%
2	LFTs	Serum bilirubin	0.8 mg/dL	0.2-1 mg/dL
		Alkaline phosphatase	146 U/L	<240 U/L
		SGOT	27 U/L	<31 U/L
		SGPT	25 U/L	<34 U/L
3	RFTs	Blood urea	23 mg/dL	<50 mg/dL
		Serum creatinine	1.02 mg/dL	0.7-1.2 mg/dL

## Discussion

In 1962, Mihalyka reported a case that may have exhibited features of DPD; however, the diagnosis could not be confirmed due to the absence of a histological description [4]. Later, in 1981, Seifert et al. detailed the clinical and histological characteristics of the condition and introduced the term "bilateral DPD" to describe this entity [3].

When this lesion involves the major salivary glands, it tends to present with a characteristic clinical pattern. Either patients report a recurring, painless swelling in the affected gland, without noticing any issues related to saliva production or structural abnormalities, which is more common, or they may experience a long-standing, non-tender enlargement of both parotid glands, sometimes persisting for several months or even years, which is less common. Recurrent parotid swelling typically begins in childhood, although clinical symptoms of the disorder may not become apparent until adulthood [5].

Batsakis et al. described the histological features of this disease in three female patients with clinical features of bilateral parotid swelling since childhood in two of those, while the third initially presented with unilateral swelling at puberty, which later developed involvement of the opposite gland in early adulthood [9]. In 1998, Garcia et al. documented the first known case in the French literature involving bilateral submandibular gland involvement [4,9]. Our patient presented with bilateral painless swelling with a history of repeated similar complaints since childhood.

Differential diagnoses of DPD include sialodochitis, chronic sialectasis, and infiltrative malignancies like lymphoma or leukemia [10,11]. Other differentials may include viral infection, granulomatous disease, or autoimmune disorder. In sialectasis, patients often present with a history of recurrent swelling, typically precipitated by meals and commonly linked to salivary stone disease. Leukemia and lymphoma have their own characteristic systemic features. Viral infections such as mumps may also lead to parotitis; however, these cases usually feature systemic symptoms and are diagnosed clinically, rarely requiring imaging. Granulomatous diseases like sarcoidosis tend to affect multiple organ systems and are usually identified through characteristic radiologic features or confirmed by biopsy. Autoimmune disorders, including Sjögren's disease, should also be considered in the differential diagnosis. However, affected individuals typically present with decreased salivary flow and additional systemic symptoms.

Although rare, polycystic parotid disease (PCD) should be included in the differential diagnosis when the following characteristics are present: (a) bilateral parotid gland enlargement in a female patient; (b) absence of systemic conditions such as Sjögren's syndrome or sarcoidosis; and (c) MRI revealing numerous small foci of low signal intensity within markedly enlarged parotid glands on precontrast T1-weighted sequences, accompanied by diffuse, prominent hyperintensity on T2-weighted sequences.

The radiographic findings correspond with the lesion's histologic characteristics, showing enlargement of the bilateral parotid gland with multiple cystic areas replacing the normal glandular tissue.

Computed tomography (CT) imaging was conducted in two previously documented cases. In one case, the CT appearance was similar to that observed on precontrast T1-weighted MRI, revealing glandular enlargement with numerous cystic spaces [12,13].

In our view, the clinical and imaging features collectively offer a distinctive profile that aids in distinguishing this condition from other pathologies. Given that sialography does not produce a specific diagnostic pattern in such cases, its utility is limited and primarily lies in excluding other potential disorders. Our case was diagnosed on an MRI.

On gross examination, these lesions typically display prominent lobulation with yellow to ivory-colored nodules that have a spongy texture [6]. Histologically, these nodules correspond to dilated ducts. While the overall glandular architecture remains intact, the lobules are expanded and largely replaced by epithelial-lined cysts, creating a characteristic honeycomb or lattice-like pattern. Scattered among the cysts are small, variably sized remnants of glandular acini [6]. The cystic spaces are lined by a mix of flattened, cuboidal, or columnar epithelium. The columnar cells feature abundant eosinophilic cytoplasm and rounded luminal borders, bearing a resemblance to apocrine cells. In some regions, bud-like epithelial proliferations are observed, while other areas display spur-like, incomplete septa. Many epithelial cells exhibit prominent cytoplasmic vacuoles containing lipid material. In certain instances, ducts open directly into the cystic spaces, and some acinar units connect with the cysts, supporting the hypothesis that these cysts originate from intercalated ducts.

Most of the cystic lumina contain flocculent, eosinophilic material along with a few scattered macrophages. Additionally, many cysts harbor eosinophilic bodies displaying concentric and radial patterns previously reported in six cases [3,5,12]. Brown et al. described amyloid-like material observed on aspiration [5]. However, an open biopsy from the same case revealed that this amyloid-like material was actually eosinophilic spheroliths [8]. These observations were comparable to those reported by Dobson and Ellis [14]. Despite the extent of involvement, there is minimal to no inflammation observed in the glands or ducts [14].

Since the lesion is benign on a microscopic level, the preferred treatment is complete surgical removal. When the parotid gland is affected, successful outcomes have been achieved through lobectomy or superficial parotidectomy [9]. In our case, the surgeon opted for total parotidectomy. Given the potential involvement of multiple salivary glands, it is important to evaluate other glandular organs such as the contralateral salivary glands, pancreas, kidneys, and spleen. Due to the limited number of reported cases, long-term follow-up is crucial to monitor for recurrence and to detect any possible involvement of additional salivary glands [4].

Genetic analysis of reported cases could reveal mutations responsible for this condition and facilitate comparison with polycystic diseases affecting other organs. Given the likelihood of bilateral gland involvement, which may require lobectomy as the treatment of choice, there is a risk of impaired salivary gland function, potentially impacting oral health.

## Conclusions

DPD of the salivary glands is a rare, non-neoplastic condition primarily affecting the parotid glands, with a notable female predominance. This case highlights a classic presentation of bilateral parotid gland enlargement in a young woman, with characteristic MRI and histopathological findings confirming the diagnosis. Awareness of DPD as a differential diagnosis for recurrent or persistent parotid swelling is essential to avoid misdiagnosis and ensure appropriate surgical management.
